# Thoracic trauma now and then: A 10 year experience from 16,773 severely injured patients

**DOI:** 10.1371/journal.pone.0186712

**Published:** 2017-10-19

**Authors:** Klemens Horst, Hagen Andruszkow, Christian D. Weber, Miguel Pishnamaz, Christian Herren, Qiao Zhi, Matthias Knobe, Rolf Lefering, Frank Hildebrand, Hans-Christoph Pape

**Affiliations:** 1 Department of Orthopaedic Trauma, RWTH Aachen University, Aachen, Germany; 2 Harald Tscherne Research Laboratory, RWTH Aachen University, Aachen, Germany; 3 IFOM - Institute for Research in Operative Medicine, Faculty of Health, Department of Medicine, Witten / Herdecke University, Cologne, Germany; 4 Department of Trauma Surgery, University Hospital Zurich, Zurich, Switzerland; Public Library of Science, FRANCE

## Abstract

**Background and purpose:**

Thoracic trauma remains to be a relevant injury to the polytraumatised patient. However, literature regarding how far changes in clinical guidelines for pre- and in-hospital trauma management and diagnostic procedures affect the outcome of multiple injured patients with severe chest injury during a long-term observation period is sparse.

**Methods:**

Multiple traumatised patients (age≥16y) documented in the TraumaRegister DGU^®^ (TR-DGU) from January 1^st^ 2005 to December 31^st^ 2014 with severe chest trauma (AIS≥3) were included in this study. Demographic data, the pattern of injury, injury severity, radiographic emergency procedures, indication for intubation, duration of mechanical ventilation, emergency surgery, occurrence of complications and mortality were evaluated per year and over time.

**Results:**

A total of 16,773 patients were analysed. The use of whole body computer tomography increased (p<0.001), while the incidence of plain x-rays decreased (p<0.001). Furthermore, incidence of AIS_Thorax_ = 3 graded injuries increased (p<0.001) while AIS_Thorax_ = 4 decreased (p<0.001). Both, rate of patients being intubated at the time of ICU admission decreased (p<0.001) and the time of mechanical ventilation decreased (p<0.001). Additionally, need for emergency surgery, lung failure, sepsis, and multi organ failure all decreased (p<0.001). However, mortality remained unchanged.

**Interpretation:**

Severity of severe chest trauma and associated complications decreased while diagnostics and treatment improved over time. However, mortality remained unchanged. Our results are in line with those expected in the context of the incidence of CT diagnostics, which has increased parallel to the clinical outcome Thus, our data demonstrate a positive trend in the treatment of patients with severe chest trauma.

## Introduction

In multiple traumatised patients, approximately 50% are affected by a serious chest injury, which continues to significantly influence the outcome in this patient cohort [1]. In this context, mortality was reported to dramatically increase in patients with thoracic trauma (up to 30%) [2]. As the lung represents a target organ for secondary damage by posttraumatic inflammation [3], lung injury contributes to the development of multiple organ failure (MOF) and therefore represents a major cause of late deaths (24%) after severe trauma [4].

However, progress in the early diagnosis and treatment of thoracic injuries has been noticed during the last decade. Computer tomography was found to reveal otherwise underestimated or overlooked injuries in the multiple injured [5], positively influence decision making in regard to operative strategies (i.e. chest tube, thoracostomy) [6–8], and guide intensive care procedures (i.e. mechanical ventilation concepts) were reported to increase patient outcome [9, 10].

However, there is consensus about the fact that thoracic trauma and its complications (i.e. sepsis, organ- and multi organ failure) remains to be of high importance [11, 12]. Due to improved diagnostic and management strategies, we hypothesised that the severity of thoracic trauma and its complications as well as mortality in patients with severe chest trauma decreased over the last decade. Furthermore we hypothesised that diagnostic procedures have changed during a 10 year period. We therefore used detailed information per year to reveal a positive long-term trend in multiple traumatised patients during the last decade.

## Materials and methods

The TraumaRegister DGU^®^ (TR-DGU) of the German Trauma Society (Deutsche Gesellschaft für Unfallchirurgie, DGU) was founded in 1993 [13]. The aim of this multi-centre database is a pseudonymised and standardised documentation of severely injured patients.

Data are collected prospectively in four consecutive time phases from the site of the accident until discharge from hospital: A) Pre-hospital phase, B) Emergency room and initial surgery, C) Intensive care unit and D) Discharge. The documentation includes detailed information on demographics, injury pattern, comorbidities, pre- and in-hospital management, course of intensive care unit, relevant laboratory findings including data on transfusion and outcome of each individual. The inclusion criteria are admission to hospital via the emergency room with subsequent ICU/ICM care or reaching the hospital with vital signs and dying before admission to the ICU.

The infrastructure for documentation, data management, and data analysis is provided by AUC—Academy for Trauma Surgery (AUC—Akademie der Unfallchirurgie GmbH), a company affiliated with the German Trauma Society. The scientific leadership is provided by the Committee on Emergency Medicine, Intensive Care and Trauma Management (Sektion NIS) of the German Trauma Society. The participating hospitals submit their pseudonymised data into a central database via a web-based application. Scientific data analysis is approved according to a peer review procedure established by Sektion NIS.

The participating hospitals are primarily located in Germany (90%), but an increasing number of hospitals from other countries contribute data as well (at the moment from Austria, Belgium, China, Finland, Luxembourg, Slovenia, Switzerland, The Netherlands, and the United Arab Emirates).

Participation in TraumaRegister DGU^®^ is voluntary. For hospitals associated with TraumaNetzwerk DGU^®^, however, the entry of at least a basic data set is obligatory for reasons of quality assurance.

The present study is in line with the publication guidelines of the TraumaRegister DGU^®^ and registered as TR-DGU project ID 2015–033.

Injuries were coded according to the Abbreviated Injury Scale (AIS, version 2005/2008, Association for the Advancement of Automotive Medicine, Barrington, IL). Until 2008, the AIS-1998 version was used. Since 2009, the TR-DGU uses a reduced version of the AIS-2005/08, where similar codes with the same severity level were merged but different severity levels were preserved. Codes from before 2009 were re-coded in this new system by preserving the severity level (except the severity changed in the AIS 2005/08 version). The severity of injuries was recorded according to the AIS as 1 (minor), 2 (moderate), 3 (severe, not life-threatening), 4 (serious, life-threatening), 5 (critical, survival uncertain), and 6 (maximum, currently untreatable).

### Inclusion and exclusion criteria

All patients treated in German hospitals, presenting with a severe thorax trauma (AIS ≥ = 3) documented in the TR-DGU from January 1^st^ 2005 to December 31^st^ 2014 were included in the present study. To adequately judge the relevance of thoracic injuries, AIS in other body regions was limited to AIS< = 3. Furthermore, all patients received intensive care treatment. Patients transferred to the reporting hospital after initial treatment in another hospital were included. However, prognostic scores could not be calculated since the initial status on admission was unknown. Primary admitted patients who had been transferred out into another hospital within 48 hours were excluded since their final outcome was unknown. Patients were analysed per year of admittance and compared over a 10 year period.

Severity of thoracic trauma was considered, and diagnostic and therapeutic procedures that are typically used in the emergency situation (plain x-rays and computer tomography (CT)) were evaluated. Therapeutic interventions included intubation, application of a chest tube, fluid therapy, administration of catecholamines and analgosedation as well as cardiopulmonary resuscitation (CPR) and need for emergency surgery. The duration of mechanical ventilation was recorded. Furthermore, the occurrence of lung failure according to the Sequential Organ Failure Assessment Score (SOFA) was analysed [14]. Organ function was considered to be inappropriate and marked as organ failure when the SOFA score was ≥3. The incidence of systemic organ impairment (sepsis and multiple organ failure (MOF)) was analysed. The diagnosis of sepsis was made according to the criteria of the American College of Chest Physicians/Society of Critical Care Medicine (ACCP-SCCM) consensus conference committee [15, 16] and MOF was defined as simultaneous failure of least two organs. Mortality was reported as in-hospital mortality. Furthermore, we used the Revised Injury Severity Score (RISC II) to predict the risk of death in severely injured patients that were primarily admitted to one of the reporting trauma centres and compared the data to the mortality rate in the same group of patients [17]. As the RISC II score is only validated for primarily admitted patients, prognosis will be performed in primary admitted patients only. Thus, patients that were secondarily transferred in, were excluded in the RISC II subgroup analysis.

### Statistics

Descriptive analysis for the 10 year period was provided for each year. Categorical variables are presented as percentages with the underlying total. Metric data were presented as mean with standard deviation (SD); in the case of skewed distribution, the median is also presented. A trend over time was evaluated with the chi-squared test in the case of categorical variables. For metric variables, a linear regression was performed, with the year of trauma as an independent predictor for the variable in question. The p-value presented is the one of the regression coefficient. A p-value < 0.01 was considered statistically significant. However, due to the large sample size in the registry, even minor differences could become statistically significant. Therefore, interpretation of results should focus on the clinical relevance rather than on significance. All statistical analyses were performed using SPSS statistical software (SPSS 22.0; IBM Inc., Armonk, NY, USA).

## Results and discussion

In total, 16,773 patients with thoracic trauma fulfilled the inclusion criteria and presented with a relevant thoracic trauma (Table 1). Of these 14,941 were primarily admitted to the reporting hospital and 1,832 were secondarily transferred in. All in all 95.4% suffered from blunt and 4.6% from penetrating injuries. The most common additional injuries (AIS 2–3) were to the extremities (24.2%), the head (18.8%) and the abdomen (10.0%). Injury mechanisms are displayed in Table 2.

**Table 1:** Demographics, injury severity (ISS), mortality and abbreviated injury score for thoracic injury (AIS_Thorax_) between 2005 and 2014.

Year	No. of patients	Age	Male	ISS	Mortality	Thorax AIS 3	Thorax AIS 4	Thorax AIS 5	Thorax AIS 6
		mean (SD)	%	mean (SD)	%	%	%	%	%
2005	561	44.4 (18.8)	78.8	21.5 (8.3)	4.8	56.7	35.5	7.1	0.7
2006	799	44.2 (19.2)	77.0	21.8 (7.5)	4.8	57.8	31.8	10.4	0.0
2007	1221	43.2 (18.8)	77.8	21.5 (7.6)	4.3	60.6	29.5	9.7	0.2
2008	1295	44.7 (19.3)	76.4	21.1 (7.3)	3.7	60.5	30.7	8.8	0.1
2009	1345	46.5 (19.8)	76.0	21.5 (7.1)	5.3	62.3	28.0	9.7	0.1
2010	1606	46.6 (19.6)	76.7	21.7 (7.7)	5.0	62.2	26.7	10.8	0.2
2011	2200	47.8 (19.9)	76.2	20.9 (7.5)	4.1	66.1	24.4	9.3	0.2
2012	2348	49.6 (19.8)	76.1	20.6 (7.7)	5.8	67.5	23.1	9.1	0.3
2013	2541	50.2 (19.9)	74.5	19.9 (7.3)	5.2	70.3	20.0	9.7	0.0
2014	2857	50.7 (20.1)	76.2	19.6 (7.3)	4.4	71.4	19.7	8.7	0.1
total	16,773	47.8 (19.9)	76.2	20.7 (7.5)	4.8	65.6	24.8	9.4	0.2
**p value**^a^		**<0.001**	**0.043**	**<0.001**	**0.44**	**<0.001**	**<0.001**	**0.70**	**0.38**

^a^ = p-value for trend over time

**Table 2:** Injury mechanisms between 2005 and 2014.

	Injury Mechanism						
	Car	Motorbike	Bicycle	Pedestrian	High Fall (>3m)	Low Fall (<3m)	Other
	%	%	%	%	%	%	%
2005	35.1	16.6	5.3	6.4	15.5	5.7	15.3
2006	35.6	16.5	4.6	6.5	16.2	6.8	13.8
2007	38.1	18.1	4.5	6.1	15.8	6.4	11.0
2008	38.5	16.7	5.5	5.7	17.4	5.7	10.5
2009	35.0	15.7	6.5	7.0	19.5	8.6	7.8
2010	38.6	16.8	5.8	5.4	18.7	8.6	6.2
2011	34.8	18.9	7.2	5.3	16.8	10.4	6.6
2012	32.8	16.4	7.3	7.1	18.4	11.4	6.4
2013	34.1	17.3	6.5	5.9	18.4	11.7	6.2
2014	30.5	18.2	8.9	5.4	17.6	13.1	6.4
total	34.7	17.3	6.7	6.0	17.7	9.8	7.9
**p value**^a^	**<0.001**	**0.28**	**<0.001**	**0.34**	**0.09**	**<0.001**	**<0.001**

^a^ = p-value for trend over time

While the rate of AIS_Thorax_ = 3 increased from 2005, incidence of severe thoracic injuries with an AIS_Thorax_ >3 decreased (Table 1).

**Fig 1:**
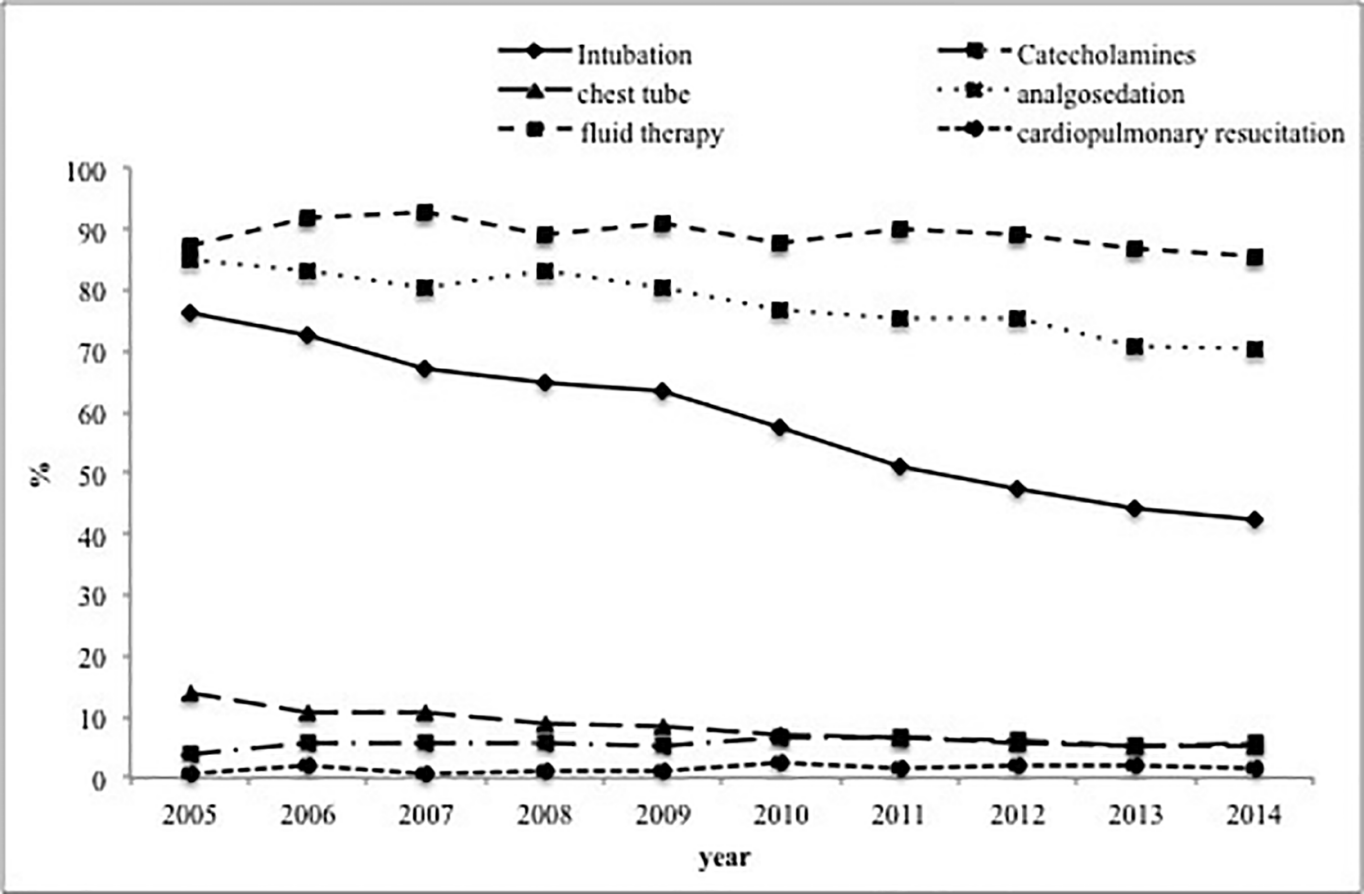
Therapeutic intervention at hospital admission in primarily admitted patients per year between 2005 and 2014.

Paralleled by a decreasing rate of severe chest injuries, diagnosed in the emergency department, the number of patients with need for intubation also decreased (p<0.001) (Table 1 and Fig 1). While 76.3% of patients were ventilated in 2005, this was only true for 42.2% of cases in 2014 (p<0.001). In accordance, patients received less analgosedation over years (2005: 84.8% vs. 2014: 70.4%, p<0.001). Duration of ventilation in those who were intubated dropped from 9.8 (SD 11.3; median 6) days in 2005 to 8.5 (SD 11.4; median 4) days in 2014 (p<0.001). While 86.1% of all multiple injured patients received emergency surgery in 2005, this accounted for 76% in 2014 (p<0.001).

The incidence of organ failure in the central nervous system (CNS), liver and kidney either showed only minimal changes or presented adverse effects (Coagulation) over the observation period. In contrast, the occurrence of organ failure in the cardiopulmonary system showed a pronounced fall (Table 3).

**Table 3:** Development of organ failure between 2005 and 2014.

	Organ Failure					
	CNS	Kidney	Liver	Coagulation	Heart /Circulation	Lung
	%	%	%	%	%	%
2005	14.8	3.1	2.0	5.5	26.0	25.8
2006	11.3	3.6	2.6	5.2	26.3	27.9
2007	14.4	4.4	3.1	5.5	25.6	23.8
2008	15.5	3.4	1.9	4.8	26.3	21.2
2009	11.7	4.2	2.5	9.4	23.1	22.0
2010	107	4.5	1.8	9.3	21.0	23.1
2011	9.1	3.8	1.6	9.1	18.4	18.3
2012	10.9	4.5	1.9	9.6	18.0	18.3
2013	10.4	3.0	1.7	7.2	17.3	16.2
2014	10.2	3.2	1.4	7.3	17.1	16.0
Total	11.3	3.8	1.9	7.7	20.3	19.6
**p value** ^a^	**<0.001**	**0.22**	**0.001**	**0.001**	**<0.001**	**<0.001**

^a^ = p-value for trend over time

**Fig 2:**
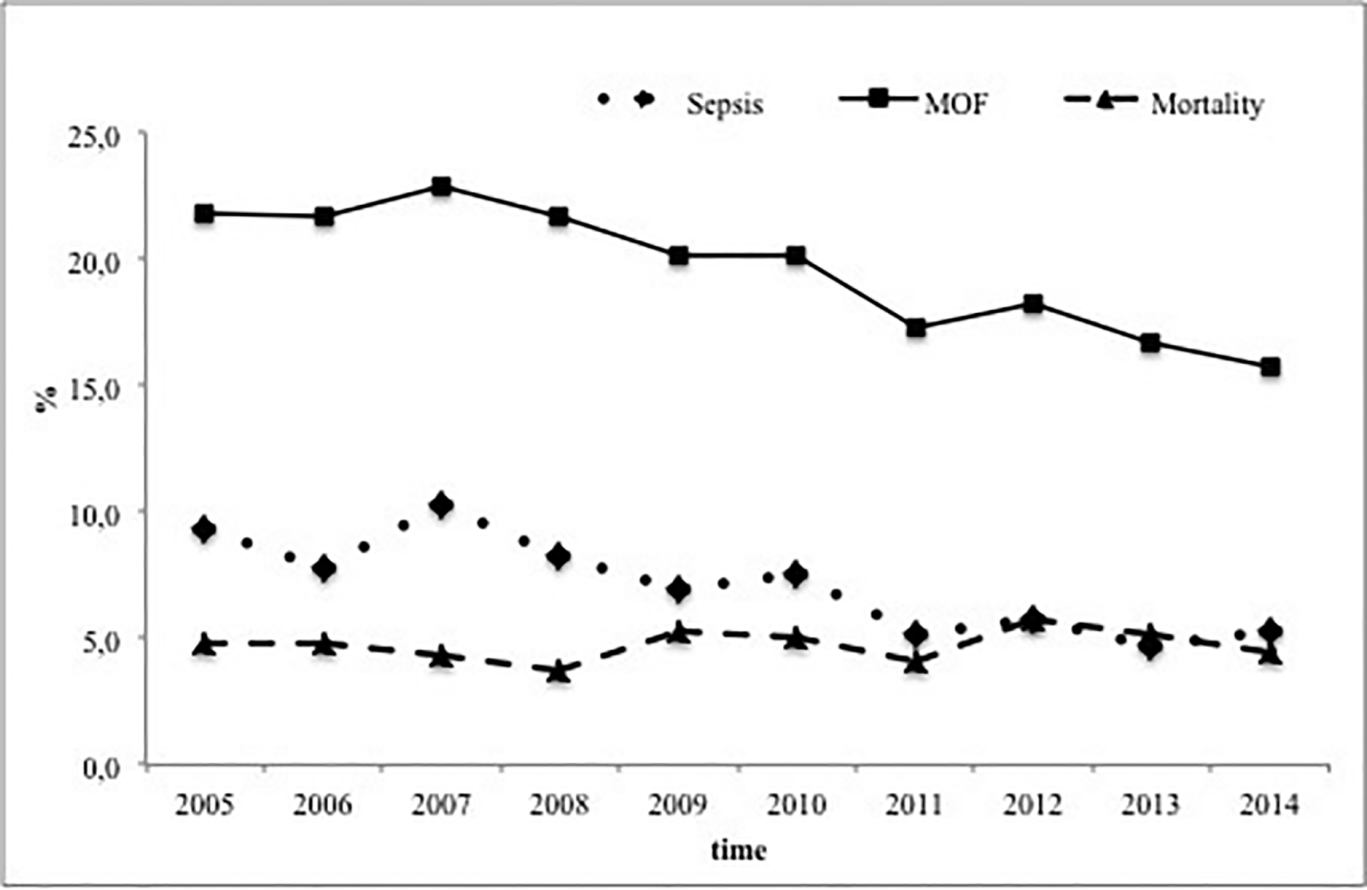
Significant decrease of sepsis, MOF and but not mortality rates between 2005 and 2014.

Also, sepsis (p<0.001) and MOF (p<0.001) rates decreased over the decade (Fig 2). The overall mortality rate was always around 5% and did not show a trend (p = 0.44) (Table 1 and Fig 2). However, subgroup analysis in primary admitted patients and complete data for RISC II analysis (n = 14,873) showed good outcome results. Predicted death rates decreased by 0.1% per year from 5.9% in 2005 to 4.7% in 2014 (p = 0.009) (Table 4). The observed mortality in this cohort was mostly lower than predicted (Table 4).

**Table 4:** Mortality, predicted death rates and standardized mortality ratio (SMR) in primary admitted patients between 2005 and 2014.

year	2005	2006	2007	2008	2009	2010	2011	2012	2013	2014	10 years	p-value
**n**	490	666	1067	1121	1183	1434	1941	2104	2294	2573	-	**-**
**mortality (%)**	5.1	4.7	4.2	3.5	5.0	5.3	4.2	5.6	5.1	4.1	4.7	**0.72**
**RISC II prognosis (%)**	5.9	5.9	5.2	5.1	5.8	6.0	5.1	5.5	5.0	4.7	5.3	**0.009**
**SMR**	0.86	0.80	0.81	0.69	0.86	0.88	0.82	1.02	1.02	0.87	-	**-**

**Fig 3:**
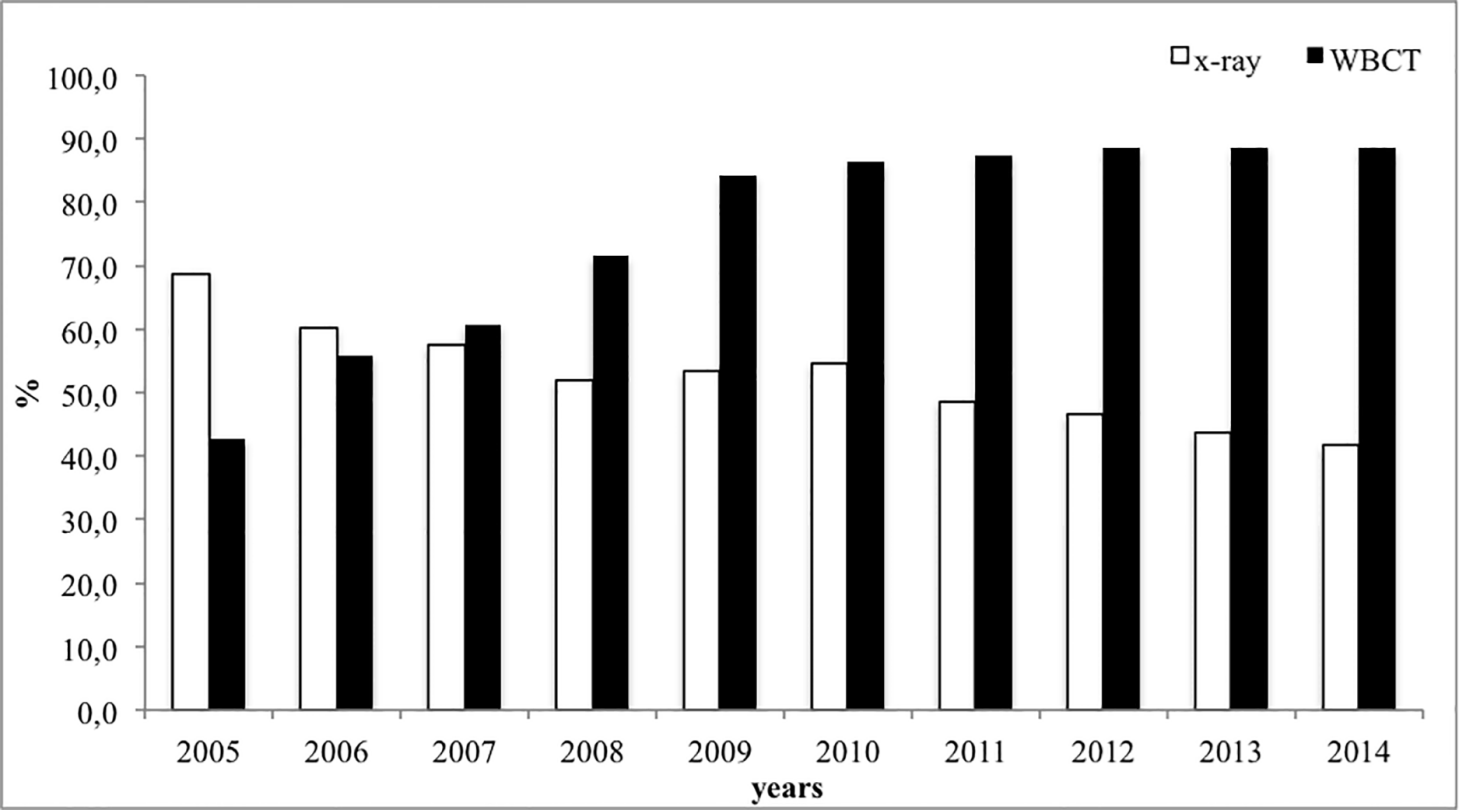
Use of plain x-rays and whole body CT (WBCT) between 2005 and 2014.

Finally, it was seen that use of whole body computer tomography (WBCT) in the diagnosis of thoracic injuries has doubled in the past 10 years (2005: 42.8% vs. 2014: 88.6%, p<0.001), while plain x-rays of the thorax have decreased from 68.8% in 2005 to 41.8% in 2014 (p<0.001) (Fig 3).

Chest trauma is one of the most important contributors to the development of complications and mortality in multiple traumatised patients. Typically blunt rather than penetrating injury mechanisms lead to injury of thoracic structures such as lung tissue, bones, vessels or the heart. Our data support findings whereupon the majority of patients present with an AIS_Thorax_ of 3 [18]. Interestingly, the number of patients being diagnosed with an AIS_Thorax_ of 3 has increased over the years, while those with an AIS_Thorax_ of 4 has decreased. In contrast, the number of patients with an AIS_Thorax_ >4 remained relatively stable. Our main results can be summarised as follows:

1. thoracic trauma severity decreased over the last decade
2. incidence of sepsis and MOF decreased significantly in patients with severe thoracic injury
3. need for intubation and length of mechanical ventilation decreased
4. the use of diagnostic procedures changed from the application of plain x-rays towards CT
5. although predicted death rates improved, mortality in patients with severe chest trauma remained unchanged.

### Organ failure and mortality

Despite sepsis, traumatic insults have been identified to contribute significantly to the development of ARDS with subsequent lung failure [19]. Insults to the lung either affect the alveolar endothelium (e.g., pneumonia, aspiration) or the microvascular endothelium (e.g., sepsis, pancreatitis, shock) [20]. However, at a distinct point, diffuse inflammation triggers further disease of the lung tissue. The inflammatory network leads to alveolar and interstitial oedema, reduced alveolar fluid clearance, impaired surfactant production and function, and lung fibrosis, which finally results in respiratory failure [20]. The release of inflammatory mediators from damaged lung tissue triggers systemic inflammation and promotes multiple organ failure, which represents a major cause of late deaths (24%) after severe trauma [4, 20, 21]. In contrast to Böhmer et al. who reported on a slight and non-significant increase of single- as well as multi-organ failure in multiple traumatised patients, the present study specifically focuses on polytraumatised patients with severe chest trauma and reveals a decreasing trend with regard to lung failure and circulatory impairment. Although lung injury was found to be associated with a pronounced inflammatory response [22, 23], recent findings by Calfee et al. who reported lower levels of plasma markers of lung epithelial and endothelial injury (ICAM-1, vWF, SP-D, and sTNFr-1) may indicate that the pathophysiology of trauma-related acute lung injury may be different from that of the broader population of lung injury patients [24]. Thus, the observed trend might be explained. Bakowitz et al. recently reported that patients with trauma-associated lung injury have not received as much investigative attention as their medical and sepsis-afflicted counterparts with ALI/ARDS, which accentuates the difference in the underlying disease [12]. Finally, the great number of clinical and experimental studies that have focused on thoracic trauma unveil the lack of knowledge in the field of thoracic trauma and inflammatory response [23, 25, 26].

In general, reports on morbidity and mortality in trauma populations are encouraging [1, 27–29]. In accordance with Ciesla et al. who reported an encouraging decrease in the progression of ARDS and MOF [30], we also found a decrease of lung failure in patients with severe thoracic trauma, demonstrating a positive trend in the treatment of patients with severe thoracic trauma. As the majority of patients with severe thoracic trauma are coded with Thorax AIS 3 [18], it may be assumed that the described improvements mainly account for this patient group rather than for the small subgroup of patients with devastating injuries to the thoracic cavity. Despite the fact that about one third of all patients with chest wall trauma is associated with pulmonary complications [31], the true mortality rate for patients with severe chest injuries is hard to evaluate as blunt chest wall trauma causes death indirectly, through pulmonary and non-pulmonary complications [32]. Furthermore, patients with severe injuries require more acute interventions, have higher rates of extra-thoracic injuries, complications, and mortality [18, 33]. Although the RISC II scores to predict death reveal a positive trend in our subgroup analysis, further studies that will illuminate pathologic mechanisms and reveal predictors for outcome in patients with severe chest trauma to improve the treatment of this specific trauma population are urgently required.

### Treatment principles

With regard to chest trauma, the need for intubation and ventilation time are valid metrics with which to measure outcome. It is well known that intubation is positively correlated with trauma severity [34]. However, various effects may have redirected the view on indication for intubation during the last few years. On the one hand, pre-hospital treatment has improved and clinical trials are starting to appear, potentially signalling a reduction in mortality and pulmonary infections based on less frequent intubations [35]. Accordingly, Hussmann et al. demonstrated that pre-hospital intubation in moderately injured trauma patients (body region AIS<3) is associated with a number of risks and should be critically weighed [36]. The authors found an elevated sepsis rate and an elevated prevalence of multi-organ failure as well as organ failure in the intubated cohort [36]. In addition, Schöneberg et al. demonstrated that intubation in severely injured patients (ISS >16, GCS <9) also does not improve survival rate [37]. Furthermore, implementation of defined indications for intubation will certainly have influenced the intubation rate during the last few years [38]. Moreover, we observed a decreased number of cases with the need for emergency surgery, which will have also influenced the decision for early intubation. This is consistent with the general reduction of injury severity in traffic accidents, as previously described [39–41], as well as improved diagnostic procedures in the emergency department [42].

With regard to mechanical ventilation, we found a decrease in ventilation time in patients with severe chest trauma. Findings reporting on mechanical ventilation time are contradictory. While some authors did not find changes with regard to mechanical ventilation time [43, 44], Probst et al. described a decrease from 11.6 days to 8.7 days during a 30 year observation period [45]. However, the majority of data are derived from general trauma populations, which were not adjusted for patients with severe chest trauma. Although the structure of the registry does not allow a detailed analysis of ventilation treatment, it may be assumed that technical innovations and established concepts (e.g. lung protective ventilation, weaning protocols) relevantly affected the time of invasive ventilation [44, 46, 47]. In this context, Silva et al. reported a reduction in days with mechanical ventilation as well as a reduced rate of re-intubation by using a specific weaning protocol [48]. However, as the duration of ventilation is also positively associated with trauma severity [49] and the occurrence of complications [50, 51], a general reduction in mechanical ventilation time should be interpreted beneficially.

### Diagnostic procedures

As already mentioned, besides a growing number of accident prevention strategies [52, 53], diagnostic and treatment procedures at hospital admission also improved [54, 55]. Against this background, we registered the increased use of computer tomography, which was found to determine trauma severity more accurately than commonly used plain x-rays [56, 57]. Accordingly, other authors also reported an increased use of CT in multiple traumatised patients during their initial assessment [58]. Although plain x-rays are not yet fully replaced by CT [59, 60], the benefit of the latter radiographic tool was underlined by Huber-Wagner et al., who reported an increased survival in haemodynamically stable and unstable major trauma patients [61]. Jiang et al. confirmed these findings in 2014 [62].

### Strengths and limitations

One of the strengths is the use of the TR-DGU as a database that summarises data from institutions committed to performing optimal trauma care. Thus, all information available in the database were documented prospectively. Furthermore, the database uses homogenous inclusion criteria by including only patients admitted through the emergency department and requiring intensive care therapy. The coding expertise is assessed by computerised plausibility assessments as well as by regular feedback to every centre. It is part of the quality assurance program involved in the certification process of TraumaNetzwerk DGU^®^, and the quality of documentation is accepted to be high [63, 64]. The high quality of registry data has also been confirmed by other authors [65, 66]. Furthermore, we only used data from German hospitals, which represents a trauma population with a majority of blunt trauma [1]. However, these results might not be representative for the whole Western European population due to, e.g., different economic and structural properties. Furthermore, we did not perform separate analysis with regard to trauma severity. Thus, it may be assumed that our findings account for the majority of patients classified with Thorax AIS 3. Also, the structure of the registry does not allow a detailed description of the airway and ventilation management that was performed and no detailed or continuous laboratory information was collected. Finally, RISC II analysis is thought to predict mortality in regard to the severity of injury [17]. As the trauma registry includes only patients being admitted to an ICU, cases of death within the emergency room and the operation theatre are not included to the analysis and thus might reduce expressiveness of a direct comparison to observed mortality.

### Conclusion

During the 10 year observation period, a reduction of morbidity was observed while mortality rates in multiple traumatised patients with severe chest trauma remained unchanged. However, diagnostic procedures improved and fewer patients were intubated. The need for emergency surgery decreased, and ventilation time as well as overall stay on the ICU were reduced, with decreasing rates of lung failure. Thus, our data revealed a positive trend in the treatment of patients with severe chest trauma based on improved diagnostic procedures and posttraumatic treatment strategies.

## Abbreviations

ACCP: American College of Chest Physicians
AIS: abbreviated injury score
CNS: central nervous system
CT: computer tomography
ISS: injury severity score
MOF: multi organ failure
RISC: revised injury severity classification
SCCM: Society of Critical Care Medicine
SOFA: sequential organ failure assessment Score
WBCT: whole body computer tomography
